# Ten Years of Medical Informatics and Standards Support for Clinical Research in an Infectious Diseases Network

**DOI:** 10.1055/s-0042-1760081

**Published:** 2023-01-11

**Authors:** Sara Mora, Barbara Giannini, Antonio Di Biagio, Giovanni Cenderello, Laura Ambra Nicolini, Lucia Taramasso, Chiara Dentone, Matteo Bassetti, Mauro Giacomini

**Affiliations:** 1 Department of Informatics, Bioengineering, Robotics and System Engineering (DIBRIS), University of Genoa, Genoa, Italy; 2 Infectious Diseases Unit, Policlinico San Martino Hospital, IRCCS for Oncology and Neuroscience, Genoa, Italy; 3 Department of Infectious Disease, IRCCS AOU San Martino IST, (DISSAL), University of Genoa, Italy; 4 Infectious Diseases Unit, ASL-1 Imperiese, Sanremo, Imperia, Italy

**Keywords:** data sharing, EHRs and systems, clinical data reuse, HL7 standards, clinical data management

## Abstract

**Background** It is 30 years since evidence-based medicine became a great support for individual clinical expertise in daily practice and scientific research. Electronic systems can be used to achieve the goal of collecting data from heterogeneous datasets and to support multicenter clinical trials. The *Ligurian Infectious Diseases Network* (LIDN) is a web-based platform for data collection and reuse originating from a regional effort and involving many professionals from different fields.

**Objectives** The objective of this work is to present an integrated system of ad hoc interfaces and tools that we use to perform pseudonymous clinical data collection, both manually and automatically, to support clinical trials.

**Methods** The project comprehends different scenarios of data collection systems, according to the degree of *information technology* of the involved centers. To be compliant with national regulations, the last developed connection is based on the standard *Clinical Document Architecture Release 2* by *Health Level 7* guidelines, interoperability is supported by the involvement of a terminology service.

**Results** Since 2011, the LIDN platform has involved more than 8,000 patients from eight different hospitals, treated or under treatment for at least one infectious disease among *human immunodeficiency virus* (HIV), *hepatitis C virus* , *severe acute respiratory syndrome coronavirus 2* , and tuberculosis. Since 2013, systems for the automatic transfer of laboratory data have been updating patients' information for three centers, daily. Direct communication was set up between the LIDN architecture and three of the main national cohorts of HIV-infected patients.

**Conclusion** The LIDN was originally developed to support clinicians involved in the project in the management of data from HIV-infected patients through a web-based tool that could be easily used in primary-care units. Then, the developed system grew modularly to respond to the specific needs that arose over a time span of more than 10 years.

## Background and Significance

It is 30 years since evidence-based medicine became a great support for individual clinical expertise in daily practice and scientific research. 1 However, this required the need to conduct several clinical trials 2 and to collect a huge amount of data. To achieve this objective, the development of systems able to support first the coordination of clinical trials and then the interoperability of heterogeneous data originating from different medical centers, 3 was necessary. These kinds of systems are indispensable not only to control the huge amount of data produced by health facilities but also to build clinical pathways, research systems, and effective public health management policies.

Since the beginning of the 2000s, the ability to describe heterogeneous data through the use of different kinds of models and connect them to the available web forms has led to the setup of web applications that facilitate the exchange of research data in many fields 4 5 6 7 8 . Multicentric research networks and clinical data management systems (CDMSs) have played an important role in data storing and management within a varying range of medical domains. 9 During the last decades of the 21st century, a large number of health-related networks have been established, such as Cancer Biomedical Informatics Grid in the United States which is a computer-based network that connects scientists and institutions with the objective of empowering the sharing of data concerning cancer. 10 At a higher level, the European Clinical Research Infrastructures Network supports multinational trials in Europe, by connecting and coordinating a great number of European national centers and networks. 11 Then, the i2b2 (Informatics for Integrating Biology and the Bedside) initiative developed a tool for data sharing and integration in precision medicine. 12 In more recent years, two other research projects, ELIXIR 13 and FAIR4Health, 14 have arisen with the aim of coordinating, integrating, and supporting the sharing of the huge amount of data generated by European research organizations during publicly funded research initiatives.

This is the scenario where, in 2011, some of the authors informally created a new research network at a regional level called the Ligurian Infectious Diseases Network (LIDN) 15 during a scientific collaboration between medical groups and bioengineers. At the very beginning, the LIDN was a web platform aimed at enabling the easy collection of human immunodeficiency virus (HIV)-infected patient data in order to conduct multicenter clinical trials at a regional level, 16 the first one involved the drug Maraviroc. 17 Medical experts manually copied data from the electronic health records (EHRs) to the LIDN through a web user interface. a During the first year, the platform supported several regional and national studies. 18 However, frequent use of the platform led to the rise of well-known problems caused by manual data input. 19 20 21 First, the huge amount of time required to insert even a minimal set of data necessary to conduct a clinical trial. Second, the randomness of errors induced by manual data imputation. Therefore, the evolution of the system was highly required. In particular, structured data such as laboratory test results were already stored in the laboratory information system (LIS) in digital format and so they could be easily read and automatically transferred to the LIDN database. Thus, the paradigm of data reuse allowed the daily update of patients' clinical data without human intervention. The authors decided to start from laboratory data since they are the largest dataset used in scientific research about infectious diseases. Once the LIDN obtained a completely updated database, we planned to exploit this system to avoid manual data entry in other databases such as Antiretroviral Resistance Cohort Analysis (ARCA), 22 Italian Cohort Naive Antiretrovirals (ICONA), 23 and Italian Coordination for the Study of Allergies and HIV Infections (CISAI). 24 These are the most important cohorts for clinical studies concerning HIV at a national level.

These first progresses in the automatization process led the authors to ask for a new approval by the Ligurian Ethics Committee (LEC), specifically for the automatic connection, which was accepted with approval number: 2/2013. This also made the architecture appealing to other diseases. In fact, in the following years, some of the authors conducted other research studies on two of the main infectious diseases present in Italy. One project by the Istituto Superiore di Sanità focused on monitoring new drugs against hepatitis C virus (HCV) and, in parallel, the continuity of care in tuberculosis (TB) patients. Finally, when the COVID-19 pandemic arose, the architecture was rapidly expanded to store data for this new class of patients as well. Specific approval by the LEC was requested and obtained (163/2020–10475).

In addition to the expansion of the group of monitored infectious diseases, the system has also evolved toward the automatic collection of clinical data from other sources. In particular in 2016, the LEC approved the connection between the LIDN and the drug delivery systems of the hospitals involved in the project. Specifically, three of the involved hospitals, the ones directly connected to the LIDN (as detailed below in section “Methods”), adopt an electronic drug delivery system. One of them adopts an individual dose-packaging system, while the other two adopt a computerized physician order entry system.

## Objectives

The objective of this work is to present an integrated system of ad hoc interfaces and tools that allows pseudonymous clinical data collection, both manually and automatically, to support clinical trials. First our system drastically reduces the time and human effort necessary to collect the data, and moreover, the level of completeness is higher. Specifically, the system retrieves from the LIS always the most complete set of available information, approved by the LEC. All the data stored in the database can be easily accessed through queries. Furthermore, our system improves the quality of data collected because unlike a human that manually copies data, it does not introduce completely random errors. 25 In addition, it is possible to improve the accuracy of a computerized system and minimize errors, while it is more difficult to work on the level of attention of a human being. In a more recent but similar platform, 26 we performed a systematic review of data extracted exploiting the same systems described here and results proving the high quality of the final content of the database will be published soon. In the near future, a similar validation will also be performed on data collected in this platform.

## Methods

### The Ligurian Infectious Diseases Network

According to the project's aim and the previously mentioned approvals received from the LEC, we collected data in a central common database in pseudonymous form and then we made them available to the medical experts, participating in the project, to conduct clinical studies.

The choices that guided the architectural development of the LIDN are described extensively. 27 From a technical perspective, the LIDN is compliant with the recommendations of the electronic source data interchange group and its approach is analog to those introduced. 28 29

**Fig. 1:**
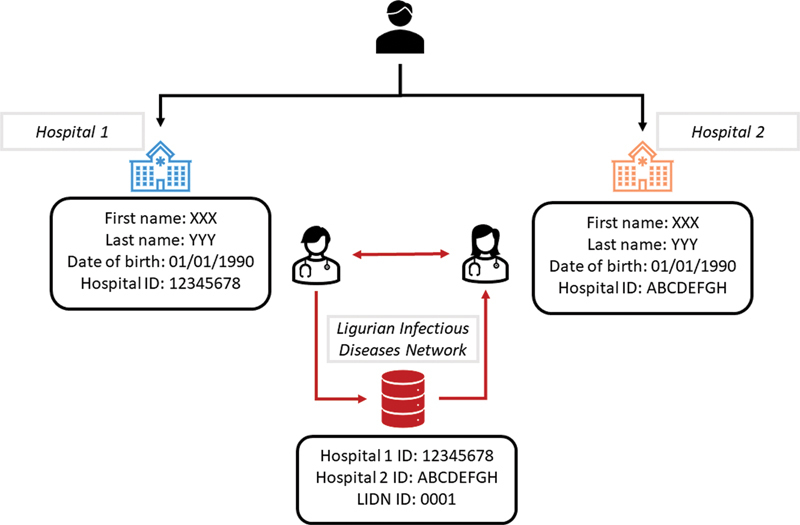
Patient's follow-up tracking system. Patient first goes to hospital 1 where the patient is identified by his personal information and a unique hospital ID. Clinician 1 decides to enroll the patient into the LIDN and the patient is assigned a unique ID specific of the LIDN. Then, the patient moves, and the patient starts going to hospital 2 where the patient is identified by personal information and another unique hospital ID. The second clinician decides to enroll the patient into the LIDN. Without any specific coordination with clinician 1 the patient would be duplicated into the LIDN because no information that could uniquely identify the patient are present into the LIDN. While, if the two clinicians coordinate themselves then into the LIDN only one record is generated, and the two hospital IDs are assigned to the same LIDN entity. LIDN, Ligurian Infectious Diseases Network.

To accomplish the General Data Protection Regulation requirements, 30 we only collected minimal sensitive information, for example, year of birth, gender, and nationality. Then, considering that most of the involved infectious diseases are chronic ones, this platform needs to identify patient events over long periods of time. Therefore, we decided to collect the patient hospital code, which does not directly identify the patient, but it helps to keep track throughout follow-ups. However, nowadays it easily occurs that a patient moves from one place to another, so it becomes necessary to track him/her through the different hospital departments. Specifically, when a patient with a preexisting diagnosis of a chronic infectious disease, for example, HIV, starts to grasp to another hospital within the one involved in the LIDN, then explicit coordination between clinicians is needed. This coordination is required as the LIDN does not contain any personal information that uniquely identifies the patient. Fig. 1 graphically explains how a patient can be tracked across multiple hospitals. This suboptimal solution has been adopted temporarily while waiting for an institutional solution to patient ID exchange. In Liguria, a platform based on the cross-reference service of the Healthcare Services Specification Project (HSSP) has been announced some time ago but is not yet available.

The platform is designed to allow the storage of different types of clinical data through specific entities called events, such as blood collections, therapy prescriptions, microbiological tests, follow-ups, diagnosis, pregnancies, immunizations, adverse events, therapeutic failures, hospitalizations, and discharges. Each event consists of different categories, for example, the blood test event includes T-cell subsets CD4 and CD8 panels, a lipid metabolism panel, a liver function panel, a metabolic panel, and a cell blood count differential panel. Each category in turn consists of several parameters, for example, the lipid panel category includes, among others, cholesterol, triglycerides, arteriosus systolic pressure, and arterial diastolic pressure. For each laboratory test result, its unit of measure and normality reference range is also stored so that in the case of multicentric clinical studies, the normalization of the laboratory test results can be achieved through the *z* -score method, therefore assuring data harmonization.

We developed the platform's database on a Microsoft SQL Server and thanks to the highly normalized structure it allows the easy addition of new parameters, categories, and event types. Highly typified data tables stored clinical results to avoid errors while saving. This means that each type of result (integer, float, string, categorical, and date) is saved into a specific database data table. Results will only be saved into the data table if they belong to the correct type. The web user-interface is developed in Microsoft .NET Framework and page visualization is standardized using Microsoft .ascx templates. These templates allow the dynamical loading of the web pages based on a specific structure defined in database tables achieving a fast personalization of the interface.

### Automatic Laboratory Information System Data Export

In multicenter clinical trials, the use of CDMS has become essential to handle the huge amount of data. Several international projects started working on this topic. In the present work, the authors describe their proposal, the LIDN that will be compared to other existing solutions in the discussion section.

Until 2013, physicians manually inserted data through a dedicated web user interface. Then, the platform evolved into its current form when the convenience of data reuse became evident as a consequence of the development of several parallel clinical trials. In particular, we implemented console applications for the automatic extraction of laboratory data directly from the LIS toward the LIDN. We decided to invest first on laboratory data because in the scenario of clinical trials on infectious diseases they are the largest dataset used.

At that time the following two possible scenarios arose, 31 based on the specific hospital level of informatization:

1. In the hospital, there was not an LIS which stored data in a digital format or there was a LIS but external agents could not obtain access to data.
2. In the hospital, there was a LIS and external authorized agents could access a specific subset of data.

Therefore, we considered two different approaches to connect new hospitals to a CDMS, such as the LIDN. Hospitals belonging to scenario 1 could take part in the LIDN only by manually inserting data through the web user interface. While those belonging to scenario 2 could exploit the data reuse paradigm. So, we set up and performed the automatic transfer of clinical data from the specific hospital LIS toward the LIDN in different ways according to the availability of services exposing patients' data and to the national regulation scenario. This choice of organizing our research platform on a regional base is guided by the Italian health care infrastructure. In particular, health care is mainly managed at a regional level and each region also developed its own health information infrastructure. 32 Some of the clinical trials supported by our platform also included data coming from other Italian regions (a complete list of clinical trials involving multiple centers is available in Table 1 ). However, these extraregional involvements are organized by medical experts that designed the specific trial depending on the scientific competencies that each center may bring to enhance it. With regard to the most recent connection, we organized laboratory data in standard documents, in accordance with the Health Level 7 Clinical Document Architecture (HL7 v3 CDA r2) guidelines. Our decision to use the CDA r2 standard was guided by national regulations that all regions must be compliant with. In particular, the Decree of the President of the Council of Ministers (DPCM) of September 2015, titled “Regulation on National Electronic Health Records (NEHR),” 33 identifies the CDA r2 as the standard schema that defines the structure of documents and messages stored in the NEHR. So, from a future perspective, if a hospital is compliant with the regulation, data could be directly retrieved from the NEHR. Since the NEHR was also founded with an explicit research aim, this new scenario will first eliminate the fundamental need to implement ad-hoc systems that read from the databases/service interfaces of each specific hospital and then it will also lead to easier connection of new centers. In particular, after the well-known COVID-19 pandemics, an instance of the NEHR has been automatically opened for everyone, and it is actually extensively used in Ligurian hospitals to store laboratory test results. In the NEHR, all data are stored in CDA r2 standard documents, so for features connections this will facilitate the automatic transfer of data. In the near future, this innovation process will also be supported by the investments allocated by the National Recovery and Resilience Plan (NRRP). Available institutional platforms will be strengthened from the standards point of view, and this will be reflected in a more satisfactory level of security and privacy. For this reason, we think that research platforms, like the one we present here, in the near future, can improve and increase their robustness and usage.

**Table 1:** Clinical trials available on the LIDN

Studies	Description	Patients	Centers
MARHIV (Maraviroc HIV)	Study on Maraviroc therapy	120	The Infectious Diseases Departments of Pietra Ligure, Sanremo, La Spezia, Torino, Alessandria, Savona, Galliera and San Martino hospitals. The Clinical Immunology department of San Martino hospital.
Long-Term	Study on HIV long term non-progressor patients	35	The Infectious Diseases Departments of San Martino and Sanremo hospitals.
ACTeA-I (Cost analysis of patients in Antiretroviral Therapy - Italy)	Analysis of the costs for patients in antiretroviral therapy	208	The Infectious Diseases Departments of Galliera, Sanremo, San Martino, La Spezia hospitals.
Vertical transmission	Study on mother-to-child transmission of HIV	51	The Infectious Diseases Departments of San Martino and Sanremo hospitals.
IANUA (Investigation on the prescribing appropriateness of antiretrovirals used in patients with HIV infection)	Study on antiretroviral prescription appropriateness in HIV	2,245	The Infectious Diseases Departments of Galliera, Sanremo, San Martino, La Spezia hospitals.
T reg (Regulatory T Lymphocytes)	Determination of regulatory T lymphocytes as a new biomarker for HIV monitoring	93	The Infectious Diseases Departments of Albenga, Galliera, Sanremo, San Martino, La Spezia hospitals.
VELA (Value of Ligurian Outcomes for the Appropriateness of HIV Therapies)	Effectiveness and sustainability strategies in the management of long-term HIV patient quality of life	275	The Infectious Diseases Departments of Albenga, Galliera, Sanremo, San Martino, La Spezia and Savona hospitals.
AIFA HCV (Italian Drug Agency pharmacovigilance project for HCV)	AIFA pharmacovigilance project for HCV therapy prescription in monoinfected and coinfected patients	1,504	The Infectious Diseases Departments of Albenga, Galliera, Sanremo, San Martino, La Spezia and Savona hospitals. The Departments of Gastroenterology and Diagnosis and Therapy of Hepatopathies of San Martino hospital.
SCUDI (Study on the costs attributable to the modification of an antiretroviral therapy)	Study on therapeutic failure in HIV patients	389	The Infectious Diseases Departments of Galliera and San Martino hospitals.
SELFY MPI (Self-Administered Multidimensional Prognostic Index)	Complete evaluation of fragility in senior HIV+ patients	157	The Infectious Diseases Departments of Galliera and San Martino hospitals.

Abbreviations: HCV, hepatitis C virus; LIDN, Ligurian Infectious Diseases Network.

Moreover, another requirement of the 2015 DPCM, cited above, is the univocal interpretation of clinical data. This is guaranteed by the presence of the CDA r2 element “Translation,” which maps each code identifying a laboratory result from the local coding system to the international vocabulary Logical Observation Identifiers Names and Codes, as requested in the specifications of the Implementation Guidelines of the CDA r2. b The involvement of a terminology service supports this process of mapping between coding systems, and it was built compliant to the standard Common Terminology Service Release 2 developed within the HSSP. c Many health care authorities highlighted the deep necessity of using automatic systems for terminology management in clinical-medical fields, among them some are settled in Italian regions. 34 35 36

## Results

Currently, the LIDN involves the infectious diseases departments of six hospitals (two in Genoa and one each in La Spezia, Pietra Ligure, Sanremo, and Savona). The network is also open to extraregional collaborations on specific projects, and currently data coming from Alessandria and Turin are also present.

### Architectures for Clinical Data Sharing

The three hospitals IRCCS AOU San Martino IST, Galliera, and Sanremo belong to the second scenario described previously in section 2.2, while the others belong to the first one. These three hospitals have different types of information systems, so we developed three different solutions to enable the sharing of clinical data. Galliera hospital offers multiple services that expose patients' clinical data, in particular, a data management service allows access to laboratory test results. After an authentication phase, data can be retrieved in two formats: XML or JSON. The request can contain some specific parameters, for example, the hospital code of the patient and starting date for the research.

On the contrary, IRCCS AOU San Martino IST and Sanremo hospitals do not share data through any services, but the information systems can give authorized external agents a read-only access to database views, built ad hoc. This way it is possible to extract the laboratory data of interest for clinical trials through the execution of simple queries.

In both cases, specific Windows Console Applications (WCAs) were developed and, to guarantee the secure read and transfer of data, we installed them within the hospital firewall. A trigger executes the console application program once each night, searching for new available laboratory tests results done during the day by registered patients. When a new patient is enrolled by clinicians into the platform, the program retrieves all historical data according to the characteristics of the disease.

As the connection between Sanremo hospital and the LIDN is the most recent one, it was built compliant with the DPCM, mentioned in section 3.2, which identifies the CDA r2 as the standard schema for clinical data sharing. Two actors are involved to make the standard connection possible: a (1) WCA which differs from the others because it organizes laboratory test results in a CDA r2 document and then sends it to a listening (2) Windows Communication Foundation service which validates the CDA r2 structure and stores the data in the LIDN database.

### Numbers of the Ligurian Infectious Diseases Network

Currently, more than 8,000 patients that have been or are still under treatment for at least one of the aforementioned infectious diseases in a center within the eight hospitals are involved in the LIDN. Considering HIV, data from about 1,900 patients from IRCCS AOU San Martino IST, about 1,200 from Galliera, and about 200 from Sanremo have been automatically monitored and updated daily using our system. The numbers are related to the hospital's catchment area, which is smaller for Sanremo hospital as it reflects a less populous area. Patients' data concerning SARS-CoV-2, since it is an acute infection, are automatically collected starting from the date of the hospital access that resulted in the first positive swab until the date on which the patient is considered cured or dies. Similarly, for data concerning HCV-infected patients, since this infectious disease evolved over time and is currently curable thanks to new treatments, clinicians are asked to insert the date of negativization or death which stops the automatic data read and transfer from the hospital database. On the contrary, since HIV is a chronic condition, patients' data are retrieved from the first positive antibodies test against HIV until the date of death or the date the patient is transferred to another hospital. Data collection rules depend on the approval by the LEC for the specific studies. So, if a study on the long-term effects of acute diseases is presented and approved, our system will be able to collect all necessary data from the day of negativization to the current date (if the patients are still in follow-up in one of the hospitals of the LIDN). In this process of automatic data retrieving, clinicians play an important role in enrolling the patients and storing the precise date of diagnosis.

**Fig. 2:**
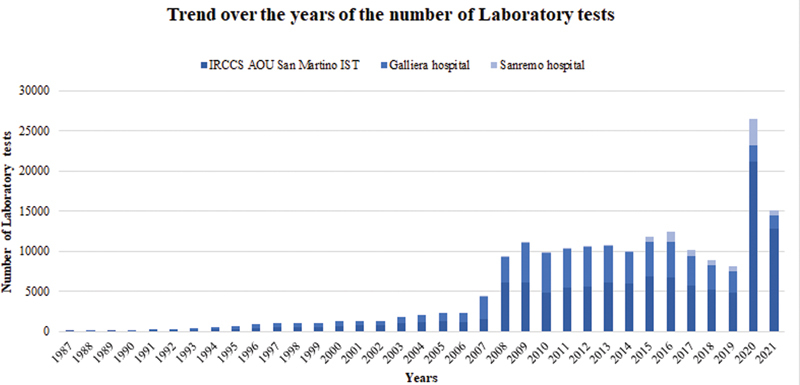
Cumulative number of tests results stored in the LIDN per year. Only centers with an automatic data connection are included in this figure so that it is possible to appreciate the increased number of data stored after 2008 (IRCCS AOU San Martino IST and Galliera hospital) and 2015 (Sanremo hospital). In 2020 the number of stored tests results is huge due to the COVID-19 pandemic, since patients have been hospitalized and so a great number of tests were done in a short period of time. COVID-19, coronavirus disease 2019; LIDN, Ligurian Infectious Diseases Network.

Fig. 2 shows the great impact that automatic data transfer had on the presence of laboratory tests available for clinical studies at Galliera hospital and IRCCS AOU San Martino IST in the LIDN, from 2008, while Sanremo hospital made laboratory test data available in the ad hoc view starting from 2015.

**Fig. 3:**
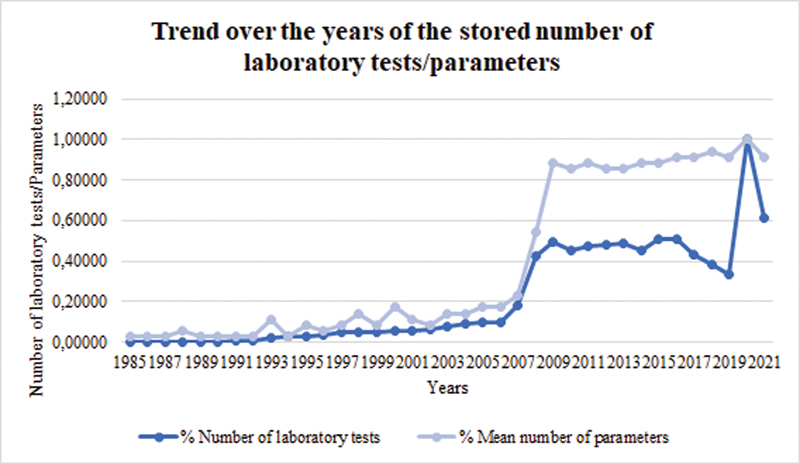
Trend over the years of cumulative number of tests results and mean number of parameters stored for each test considering IRCCS AOU San Martino IST, Galliera hospital, and Sanremo hospital. In 2020 there is a peak considering the curve of laboratory tests and the other curve reaches its maximum level, as a consequence of the COVID-19 pandemic. Patients were hospitalized so it meant a higher number of tests and new parameters in addition to those of HIV were stored, for example, emogas analysis. COVID-19, coronavirus disease 2019.

So, the differences between automatic and manual storing of data can be quantitatively evaluated considering the cumulative number of laboratory tests stored per year and the mean number of parameters collected per single laboratory test. Fig. 3 shows these two quantities, calculated for the three automatically connected centers: IRCCS AOU San Martino IST, Galliera hospital, and Sanremo hospital.

To obtain a report of laboratory activities without automatic data transfer for all patients involved in a clinical trial, with a number of collected parameters as high as the one produced by the LIDN, a huge effort would be necessary from a timing point of view in order to have a wide range of data. For example, when compiling an electronic case report form, some of the parameters to be collected can be flagged as optional. Using an automatic system, the effort necessary to transfer the complete set of all the available data that are required or just the minimum set of mandatory ones is the same, but this is not true when the data imputation is performed manually. So, when an automatic transfer system is used, the set of available data is always as wide as possible. Our system, which automatically extracts and transfers pseudonymous data from the EHRs toward the CDMS, remarkably reduced the time necessary to collect a considerable subset of the clinical data necessary to conduct research activities. In order to guarantee the high quality of our data, we periodically perform a manual validation by comparing the information stored in our database directly with those present in the LISs. Two of the main criticalities that arose during the 10 years of activity and that led to a sudden lack of information are (1) the changes in the names and codes that identify the laboratory analysis within the hospital database and this problem further highlighted the need of using standard vocabularies; (2) the transition from decimal notation to scientific notation when reporting the results of some laboratory analyses. The presented platform supported the 10 local studies listed in Table 1 17 37 38 39 40 41 each of them with its specific interface.

The increased speed of collecting high-quality data resulted in an increased frequency of publication by supporting the comparison between the different antiretroviral treatments and the periodical data update between the LIDN and the main cohorts at the national level. Two examples of important and innovative studies conducted on the amount of data available in our platforms are shown. 42 43 The first one investigates the comparison between “no treatment” disease progression costs and the economic impact of regimens based on Sofosbuvir (SOF) on a population of HIV/HCV-coinfected patients. The second one provides one of the first experiences studying the changes in lipid profile, CD4, and creatinine after switching from a combination therapy with tenofovir disoproxil fumarate to a combination therapy with tenofovir alafenamide.

### Connections to National Databases

Once a completely updated database is obtained, it could be reused to export reliable information toward national databases: ARCA, ICONA, and CISAI. At present, almost 1,300 patients involved in the LIDN are also enrolled in ARCA, 700 in CISAI, and more than 700 in ICONA. Considering ARCA and CISAI, authors set up a direct collaboration that allows an automatic update of data. On the contrary, ICONA is connected to an offline procedure based on Excel files derived from shared predefined templates.

Regarding the connection between the LIDN and the ARCA and CISAI databases, it is established at an analytical level. So, it is possible to create a complete description of the patient because the semantics of the arrival database is known. Thanks to this, all information about the single patient can be translated from the local vocabulary to the arrival database semantics in a stable way. Specifically, a WCA was developed to fill the ARCA database with the records of the LIDN patients, in particular with CD4+ cell counts, HIV RNA copies/mL, and genetic sequence data. The CISAI cohort, together with HIV, also involves HCV-infected subjects and collects data about both these infectious diseases.

### Additional Tools of the Web User Interface

**Fig. 4:**
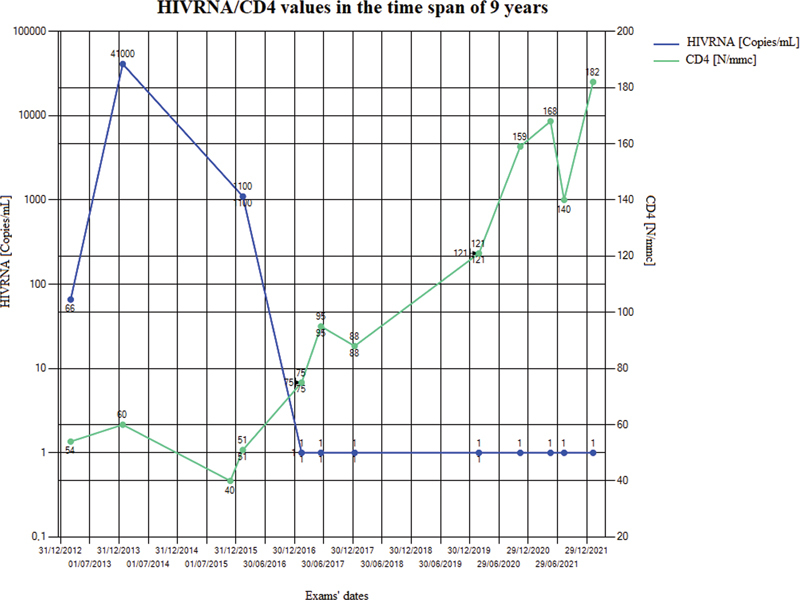
Trend over the years of HIVRNA and CD4 values for a randomly selected patient within the LIDN. The two scales have different dimension orders, the HIVRNA scale is logarithmic and values corresponding to 1 are below the threshold, while the CD4 scale is linear. LIDN, Ligurian Infectious Diseases Network.

Data stored in the database can be used to produce statistical reports and charts to visualize continuously updated information. Pie and point-line charts, histograms, and tables display absolute, relative, and percentage frequencies of data for epidemiological studies. It is also useful to monitor complicated cases because clear and synthetic information is displayed, for example, in Fig. 4 all CD4 and HIVRNA values for a randomly selected patient are displayed over a time span of 9 years. To export a selected set of information to predefined spreadsheet files, a flexible and optimized extraction tool was set up. The user can customize the following information: patient, time range, and parameters of interest.

The web interface also allows clinicians to calculate some important clinical scores, for example, Glasgow coma scale, glomerular filtrate, etc., but we decided not to store results in the database to reduce data redundancy. Other more complex scores, such as viremia copy years, can be requested by clinicians and provided in the process of data extraction for specific clinical trials.

## Discussion

**Fig. 5:**
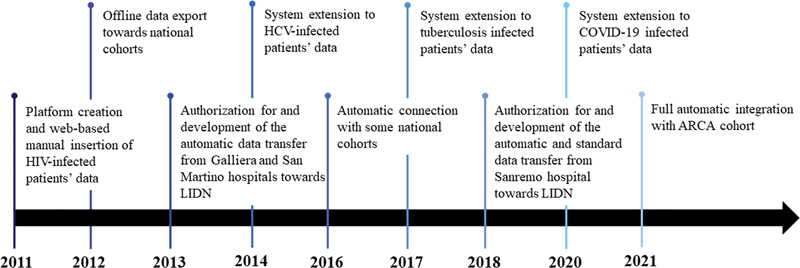
Timeline summarizing the 10-years evolution of our system, from 2011 to current.

In the present manuscript, we described the results of our work which involved many professionals from different fields for more than 10 years and whose main aim was to support infectious diseases clinical trials. We developed a modular system which grew in order to respond to the specific needs that appeared in these 10 years, for example, COVID-19 pandemics, as shown in Fig. 5 .

By describing the history of our platform development, we take the opportunity to highlight the critical issue of lack of funding for infrastructures, like ours, which are fundamental in establishing efficient clinical data management. The lack of funding was recognized by Italian government who, in the NRRP, identified as of primary importance the objective of professional management of health care data. 44

During the 10-year period, the platform was mainly developed and managed by PhD students with minimal funding and to date, the platform has survived on residual funds from the clinical trials themselves. We hope that our findings will encourage more clinical trial groups to include data management plans alongside proposal submissions. The platform staff (medical experts and one bioengineer) played an important role in the supervision and quality assessment of the interventions performed by the PhD and postgraduate students. The natural transition from one student to another produced periodical checkups of the system, and students and staff formed a deep understanding of the platform, allowing them to look for and identify possible criticalities, for example, due to the introduction of new technologies and the development of new branches.

When we started in 2011, there were no known worldwide systems with this purpose of supporting clinical trials, but at present we know that in the following years, systems with this aim grew and nowadays some of them are internationally adopted. In this discussion section, we want to compare our work with two worldwide well-known systems, Research Electronic Data Capture (REDCap) and i2b2, and we want to motivate the decision to continue using our system instead of adopting a new one. REDCap is a web-based system first released in 2004 by the Vanderbilt University with the aim of creating databases and projects for clinical trials. The main advantage is that users can quickly develop and customize databases and projects for study requirements, even though they are not programmers. Users can access projects from any device, they only need an internet connection, and they can export all data (all records and fields) in many different formats, one of which is the standard format “CDISC ODM”. The main disadvantages are that changes in the database or project structure should be approved by the informatics core and certain revisions may not be allowed; data should be collected separately for each project, and even though the REDCap system also made available some API's for the automatic data transfer from a specific EHR to the REDCap project, this feature requires more in-depth technical competences. 45 46 Recent papers, found in the literature, reported that other groups stepped back from the REDCap application to custom solutions due to specific limitations they found. 46 47

The i2b2 is a clinical data warehousing and analytics research platform that was first released in 2007, and its main aim is to create a cost-effective and efficient system for clinical and translational research. 48 It uses a simple data model that is similar to the *Event-Attribute-Value* approach and ontologies to translate concepts to local codes. The i2b2 query tool provides users with an instrument for easy cohort design and exports data in various formats compatible with many analytics tools. The main advantage of the use of i2b2 in multicenter clinical trials is that it provides an information model that once mapped to the local models can be used for several studies on different topics. In this way, it is not necessary to map data to a new information model each time we want to take part with a different cohort. 49 The main limitation is that i2b2 only allows the importation of bulk data through CSV files, many ad-hoc services and APIs have been developed within separate research projects 50 51 while others suggest the use of medical standard formats, for example, CDISC ODM. 52

The system we present is similar to REDCap, even regarding the presence of an automatic data transfer system, but on a smaller scale. In addition, the presence of a multidisciplinary team relieves clinicians from several technical issues. We think that it is not worth stopping the use of our system to build everything from scratch again. During the COVID-19 pandemic, the authors collaborated on a cardiological clinical study organized with the REDCap platform. Taking into consideration the emergency situation and the need to collect data in the shortest time possible, the authors used a predefined template to transfer data, but a future objective is to make this connection more efficient, also from a timing point of view, setting up an automatic system. To improve the semantic connection between the two systems, we expect to use the new standard ISO: 23903:2021, d in addition to the HL7 standards and the above-mentioned terminology system. This new standard is devoted to the definition of data interchange through a more profound knowledge of the data models of the sending and receiving systems.

Similar to i2b2, we exploited the data reuse concept, so that once retrieved, data can be used for several clinical trials, it is only necessary to change the queries. Then, we provide clinicians with a set of tools to evaluate patients' follow-up, for example, through the graphical visualization of the trend over time of some laboratory test results. An advantage of an “in house” system is the more efficient building of specific rules for data extraction due to the deep knowledge of the database structure. So, we have a direct access to data instead of always using the system interfaces.

The major limitation of our system is that a technician must manage it. The interface is very simple from a graphical point of view, easy to use, and specific for regular users (who always do the same operation). If, on the contrary, clinicians want to carry out more complex processing, they should request that a technician performs the changes because they cannot autonomously perform operations such as modify a page and consequently the collected parameters. The procedures illustrated in this paper do not all belong to a routine process, so they should be followed by a person with a deep knowledge of database management and whose background allows them to interpret medical requests. Therefore, we believe that the most focused figure that could assume this role is the bioengineer with a good knowledge of medical informatics. In recent years, our group also developed a university spin-off which, in addition to other projects centered mainly on the use of standards in medical informatics and planned a reengineering process of the platform in order to insert it at least in the national market. However, until now, no relevant economic evidence was found. This is due to the presence of several barriers that should be overcome in terms of finance, dissemination of standards, misperception of privacy by hospital staff, etc. On the contrary, the main favorable factors for the implementation, development, and diffusion of this kind of platform for scientific research are the laws related to the NEHR and the growing propension of medical experts toward the digitalization of health.

In our laboratory PhD students and other trainees are now applying machine learning and natural language processing. 53 54 55 It will be quite interesting and relatively easy to extract and organize these data to verify clinical and diagnostic hypotheses given a large number of data and taking inspiration from similar works already present in the literature. 56 57 58

## Conclusion

In conclusion, we think that we should keep our system, but in the following years, we will arrange a set of interfaces and services (hopefully standard based) to be ready to collaborate with other systems.

The original objective of the LIDN was to develop a web-based tool that allowed clinicians in primary-care units and specific infectious diseases wards to collect and manage data of HIV-infected patients both for patient care and scientific research. In this paper, the authors want to present the evolution over time of the original plan with the development of an automatic data transfer system first from the hospitals EHRs toward the LIDN and then from the LIDN toward the main HIV national cohorts. This process of data reuse is essential to avoid errors due to manual data input, and it remarkably reduces human effort and the time necessary to collect clinical data for research purposes. It also guarantees the high quality of the collected data. Moreover, the paper presents the evolution of the platform that at present also includes data from patients affected by other infectious diseases like HCV, TB, and more recently SARS-CoV-2.

The implemented solution, thanks to the use of medical standards, could easily be further expanded in the future by adding new regional or extraregional hospitals.

## Clinical Relevance Statement

The LIDN platform simplifies the management of clinical trials thanks to its web interface that allows smart visualization of a patient's clinical information through statistical elaborations, charts, and a user-friendly customizable query tool. Moreover, the addition of the described tool that automatically extracts data from hospital EHRs allows the management of a greater number of patients; in fact, after the enrollment of a new patient, all his clinical data are available on the platform without human effort, with continuous updating.

## Multiple-Choice Questions

1. Which is the appropriate standard for document definition and exchange according to the DPCM “Regulation on National Electronic Health Records”?
  - Clinical Document Architecture Release 2
  - Common Terminology Service Release 2
  - LOINC
  - Clinical Data Management System
  **Correct Answer:** The correct answer is option a. The CDA R2 is a standard architecture suitable for clinical document exchange and it is based on the Reference Information Model (RIM) of HL7 V3.
2. Which of the following is a possible connection to the presented system?
  - Only by CDA
  - Only manually through the interface
  - Only by not standard web services
  - Either by CDA or by manual data insert or by web services
  **Correct Answer:** The correct answer is option d. The authors developed specific interfaces for all three possible connections based on the different IT levels of the involved centers. The most appropriate one is the standard connection through CDA R2.

## Footnotes

a
LIDN homepage can be reached at: https://www.reteligurehiv.it/ .

b
Implementation Guidelines of CDA R2 “Laboratory medicine report” can be available at: https://www.fascicolosanitario.gov.it/sites/default/files/public/media/Specifiche%20HL7%20CDA%20R2_RefertoMedicinaLab-v1.1-S.pdf .

c https://www.omg.org/spec/company/healthcare-services-specification-project-(hssp)/About-healthcare-services-specification-project-(hssp)/ .

d
*https://www.iso.org/standard/77337.html* *.*
